# Classification of *Neisseria meningitidis* genomes with a bag-of-words approach and machine learning

**DOI:** 10.1016/j.isci.2024.109257

**Published:** 2024-02-16

**Authors:** Marco Podda, Simone Bonechi, Andrea Palladino, Mattia Scaramuzzino, Alessandro Brozzi, Guglielmo Roma, Alessandro Muzzi, Corrado Priami, Alina Sîrbu, Margherita Bodini

**Affiliations:** 1 Vaccines Discovery Data Sciences, GSK Vaccines, GSK, 53100 Siena, Italy; 2 Department of Computer Science, University of Pisa, 56127 Pisa, Italy

**Keywords:** Microbial genomics, Classification of bioinformatical subject, Machine learning

## Abstract

Whole genome sequencing of bacteria is important to enable strain classification. Using entire genomes as an input to machine learning (ML) models would allow rapid classification of strains while using information from multiple genetic elements. We developed a “bag-of-words” approach to encode, using SentencePiece or k-mer tokenization, entire bacterial genomes and analyze these with ML. Initial model selection identified SentencePiece with 8,000 and 32,000 words as the best approach for genome tokenization. We then classified in *Neisseria meningitidis* genomes the capsule B group genotype with 99.6% accuracy and the multifactor invasive phenotype with 90.2% accuracy, in an independent test set. Subsequently, in silico knockouts of 2,808 genes confirmed that the ML model predictions aligned with our current understanding of the underlying biology. To our knowledge, this is the first ML method using entire bacterial genomes to classify strains and identify genes considered relevant by the classifier.

## Highlights

• We recoded bacterial genomes as words and classified them using machine learning
• We tested the approach by predicting the type of bacterial capsule and invasiveness
• Predictions were accurate: 99.6% for bacterial capsule type, 90.2% for invasiveness
• This can improve tasks like monitoring antibiotic resistance and disease outbreaks

## Graphical abstract

**Figure:**
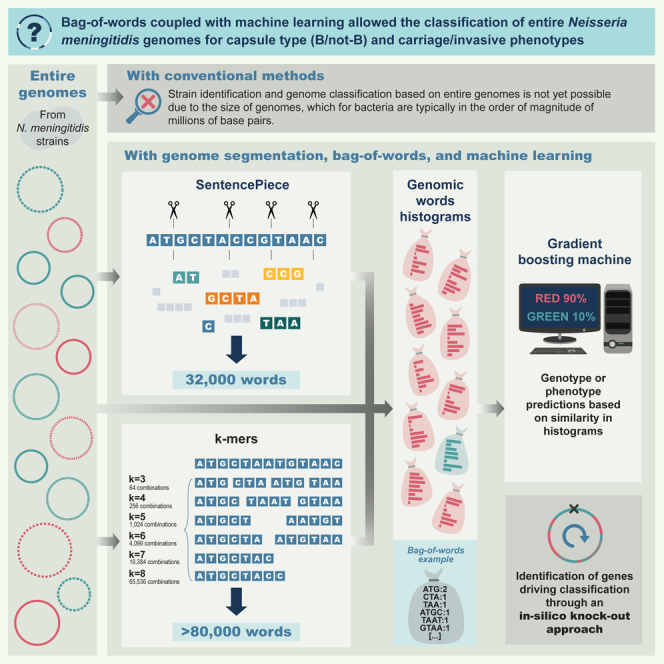

## Introduction

Whole genome sequencing of bacteria has many applications, including strain characterization, population genomics, monitoring antibiotic resistance, and outbreak investigation. The number of bacterial genomes stored in public archives (>1.2 million^1^^,^^2^ on March 9, 2023) is increasing exponentially due to decreasing costs and the higher resolution of new technologies.^1^^,^^3^^,^^4^ However, despite the tremendous number of bacterial genomes available, it is not yet possible to use entire genomes for classification.

Improvements in machine learning (ML) models for sequence analysis have enabled the identification of specific genomic traits in humans and mice.^5^^,^^6^^,^^7^ Currently, the best-in-class classifiers enable the prediction of transcription factor binding sites, splice sites, and gene expression; they also support variant calling and consensus sequence correction.^5^^,^^6^^,^^7^^,^^8^^,^^9^^,^^10^ In the future, we anticipate that ML will also provide statistical approaches for the fine-mapping of data from genome-wide association studies (GWAS) and expression quantitative trait loci (eQTL) studies,^11^ predict the impact of non-coding variants on disease,^12^^,^^13^ and predict the regulatory activity of sequences based on cross-species data.^14^ The greatest challenge related to human and murine genomes, as well as other mammalian genomes, is the length of the sequence provided as the input to the classifier: 3.055 and 2.5 billion base pairs (bp), respectively, for humans and mice.^15^^,^^16^

The smaller size of bacterial genomes, from 0.6 to 8 million bp (Mbp),^17^ makes them more manageable than mammalian ones; for example, the *Neisseria meningitidis* genome contains about 2.3 Mbp.^18^^,^^19^ Recent methods have been proposed to input entire prokaryotic genomes into deep neural networks. However, deep learning methods cannot handle extremely long sequences such as bacterial genomes. At the same time, the number of genomic sequences available is rapidly increasing, albeit not at the same pace as observed for human genomes, to the point that we can now use them for classification purposes.

In addition to difficulties due to the genome size, genome assemblies obtained using short-read sequencing techniques are difficult for ML models to handle because the genome is divided into contigs of which the direction is unknown and which can be incomplete or redundant. This variability makes it even more challenging to use sequential approaches unless multiple alignment is applied; however, multiple alignment is computationally too demanding as the number of genomes in public repositories increases. Furthermore, bacterial genomes exhibit great variability, including accessory genes not shared by the whole population,^20^ making it difficult to understand which subsequences should be compared among different genomes.

Comparative genomic methods have been developed to tackle these challenges and enable the use of entire genomes for classification purposes. One of these is the phyletic patterns approach that generates a binary presentation (a series of 1s and 0s) of the presence or absence of genes in different genomes and then compares the binary presentations.^21^ The phyletic patterns approach is useful for analyzing prokaryote evolution^22^ or identifying alien genes in a genome.^23^ Another comparative genomic method is the fuzzy profile method that generates profiles based on sets of genes present in a specific genome and compares these sets between genomes.^24^ Both methods have a low resolution that does not consider subtle changes in genes that can greatly affect gene function.

Many comparative genomic methods have been developed that do not focus on the presence or absence of genes or sets of genes, but instead use entire genomic sequences for alignment-free comparisons. These are mainly *word-based methods*, based on the frequencies of subsequences of a defined length, and *information theory-based methods*, based on the informational content of full-length sequences.^25^ An example of a word-based method is the comparison of prokaryotic genomes using k-mers to reconstruct phenetic trees.^26^ An example of an information theory-based method is the comparison of relative information between sequences using Lempel–Ziv complexity for building phylogenetic trees.^27^^,^^28^ None of the available alignment-free tools use whole genomes to predict phenotypes.

We developed a comparative genomic method based on the hypothesis that the frequency of genomic subsequences within a genome (and within a collection of genomes) could be used to predict a phenotype of interest. Inspired by SentencePiece (SP), which was developed to identify words in non-segmented languages like Chinese, Korean, and Japanese, we extracted the frequency information. Hereto, we applied tokenization (replacing subsequences with token words) and a “bag-of-words” approach (counting the frequencies of the token words) to encode bacterial genomes in their entirety and use them as inputs to standard ML methods for classification. The bag-of-words approach does not require position information, thus allowing for handling whole genomes without the need for alignment or assembly. In contrast to existing approaches, this approach would allow for rapid classification while using all information provided by multiple, possibly distant, genetic elements as well as from intergenic regions, and not being hampered by variability in gene presence or short sequence reads.

Two text tokenizers were chosen to create the vocabularies: SP and a k-mer representation. SP is an unsupervised text tokenizer mainly used in text generation systems based on neural networks where the vocabulary size is predetermined.^29^ SP allowed end-to-end English-Japanese translation, with performances similar to ML algorithms, thus overcoming the challenges of non-segmented languages. Similarly, we applied SP to genomic sequences as uninterrupted texts. The k-mer approach segments the sequences into fixed-size words and is the basis of many tools for classifying long sequences.^30^^,^^31^^,^^32^^,^^33^ The k-mer approach creates vocabularies of fixed dimensions in which all possible words with a certain number of characters are included.^34^^,^^35^^,^^36^ After creating the vocabularies with either SP or k-mer tokenization, the bag-of-words method counts the occurrence of each word (the bag-of-words) in the genome, regardless of relative position, direction, or order.

Our main objective was to determine whether the bag-of-words approach is suitable for classifying phenotypes of *N*. *meningitidis* genomes. For this, two classification tasks were set. The first was to characterize a purely genetic problem, the capsule type classification, and the second was the multifactor problem of invasive phenotype classification. The main difference between our approach and sequential approaches is that we did not consider positional information due to the unknown direction of the contigs; this is not feasible for bacterial genomes, as it would require a computationally expensive genome alignment. The secondary objective was to determine whether our ML model is really learning, by studying genes predicted by the model to be important for invasiveness and determining whether these genes are known virulence factor (VF) genes or could indeed be new VF genes based on the genes’ known biological functions.

## Results

### Model selection and evaluation

Byte pair encoding was used to create token vocabularies from a corpus of sequences. Hereto, an initial vocabulary was created where tokens were single characters, the most frequent pair of adjacent tokens was then merged into a new token, which was added to the vocabulary. All the occurrences of the two merged tokens were then replaced by the new token. This procedure was iterated to expand the vocabulary until a desired size was reached. For both capsule and disease classification tasks, experiments were performed with six variants of SP and six variants of k-mer approaches to represent an *N. meningitidis* genome as a bag-of-words. For each variant, 50 hyperparameter combinations were trained on the training set, and their performances were validated on the validation set. Both approaches exhibited high accuracy; increased accuracy was observed with increasing vocabulary size.

**Figure 1:**
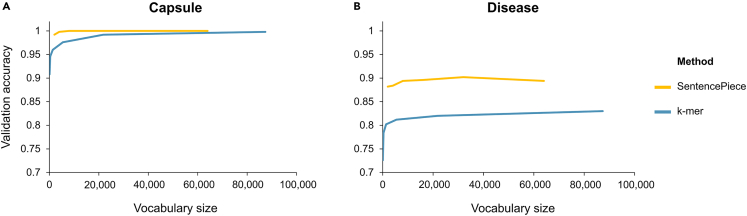
Effect of vocabulary size on validation accuracy Plots of validation accuracy when different vocabulary sizes were used in the k-mer and SentencePiece approaches. For the k-mer approach the genome was segmented in k-mers: only 3-mers (vocabulary size: 64), 3- and 4-mers (vocabulary size: 320), 3- to 5-mers (vocabulary size: 1,344), 3- to 6-mers (vocabulary size: 5,400), 3- to 7-mers (vocabulary size 21,824), and 3- to 8-mers (vocabulary size: 87,360). For the SentencePiece approach, six vocabulary sizes were evaluated: 2k, 4k, 8k, 16k, 32k, and 64k. See also Table S1. (A) Classification accuracies obtained with the test set for the capsule group classification. The blue line depicts the k-mer approach and the yellow line the SentencePiece approach. (B) Classification accuracies obtained with the test set for disease classification. The y axis depicts the accuracy score, the x axis shows the vocabulary size.

For the capsule task, the SP approach with 8k, 16k, 32k, and 64k words all returned the highest possible validation accuracy (100%), while the k-mers approach only had a high validation accuracy when using an order of magnitude more features (vocabulary size 87,360) compared with the 8k SP model (Figure 1A; Table S1). Therefore, we decided to use the SP approach with an 8k vocabulary size for independent evaluation and subsequent experiments. When using an independent test set, the 8k SP model scored 99.6% accuracy (Table 1).

**Table 1:** Capsule and disease classification performance of the SP approach on the independent test set

Task	Strategy	Number of features	Accuracy	AUROC
Capsule classification	SP	8,000	0.996	1.000
Disease classification	SP	32,000	0.902	0.968

Threshold used: 0.5. AUROC, area under the ROC curve; ROC, receiver operating characteristic; SP, SentencePiece

Similarly, in the disease task, the SP approach with 32k words had the highest validation accuracy (90.2%), while all k-mer models performed poorly in comparison (Figure 1B; Table S1). Therefore, the SP approach with a 32k vocabulary size was selected for independent evaluation and subsequent experiments. When using an independent test set, this approach scored 90.2% accuracy (Table 1).

### Capsule locus removal resulted in a capsule group prediction change

**Figure 2:**
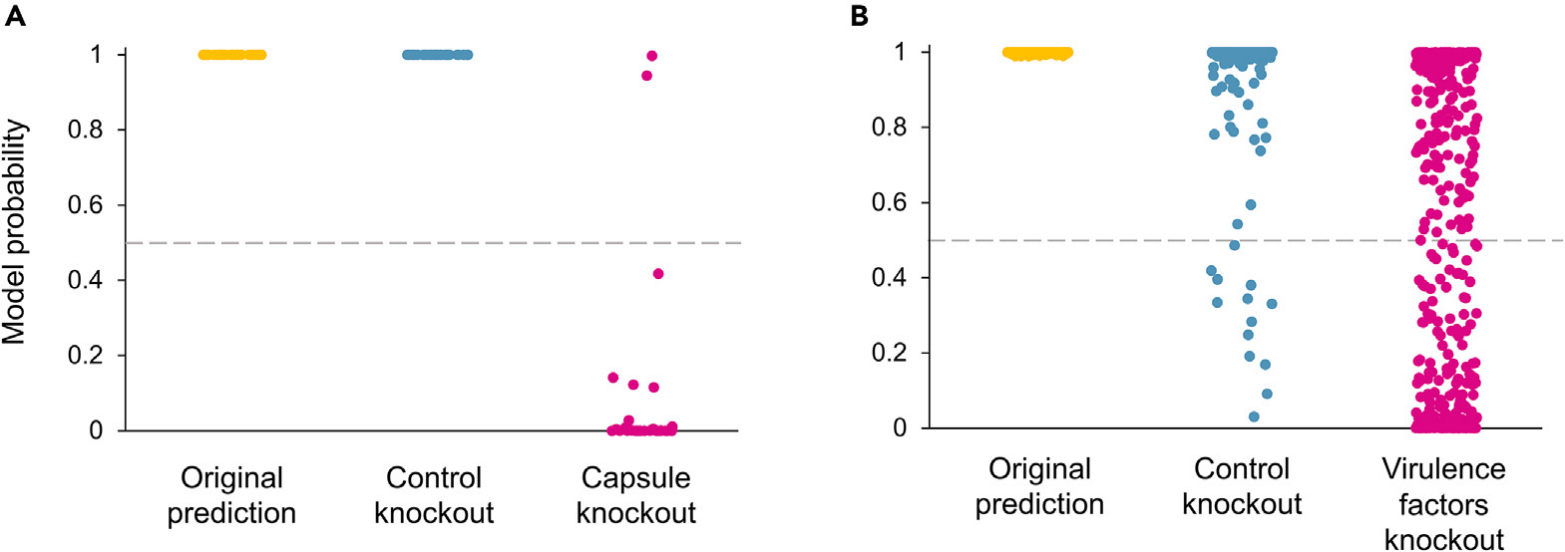
Prediction change of type B capsule or disease phenotype after knockout In silico knockouts were performed to evaluate the prediction of classification of either capsule or disease phenotype. (A) 32 genomes of strains for which initial classification predicted a type B capsule with high confidence (original prediction, in yellow) were selected. In silico knockout of the entire capsule locus (capsule knockout, in magenta) changed the prediction of the type B capsule to a non-type B capsule for 30 of the 32 strains (93.8%). In silico knockout of a random contig of the same size (control knockout, in blue) did not change the prediction of the type B capsule for any of the strains (0.0%). (B) 436 genomes of strains for which initial classification predicted an invasive phenotype with high confidence (original prediction, in yellow) were selected. In silico knockout of 155 virulence factor (VF) genes (VF knockout, in magenta) changed the prediction of the invasive phenotype to a carrier phenotype for 230 of the 436 strains (52.8%). In silico knockout of random sequences of the same size (control knockout, in blue) changed the prediction of the invasive phenotype to a carrier phenotype for 13 of the 436 strains (3.0%). Each dot represents one strain. The horizontal dashed line represents the prediction threshold of 0.5.

In *N. meningitidis*, all capsule genes are located in the capsule locus.^37^ In silico knockout of the entire capsule locus (mean number of bases removed was 25,663 bp +/− 697 bp) was performed on 32 genomes of strains for which initial classification predicted a type B capsule with a high level of confidence (≥0.99). Following this knockout, ML classification, that was optimized and trained by lightGBM, was applied to predict phenotypes. ML classification with the SP 8k approach found that most strains (n = 30/32, 93.8%) were now predicted to have a non-B capsule type (Figure 2A). In these 32 strains, the mean probability of having a type B capsule went from 1.000 (+/− 2.16E-6) before to 0.088 (+/− 0.245) after the knockout.

As a control, the same number of bp as the capsule locus were removed from the 32 genomes but from randomly chosen contigs. Following this removal, ML classification found that none (n = 0/32, 0.0%) of these strains were predicted to have a non-B capsule type (Figure 2A). In these 32 strains, the mean probability of having a type B capsule went from 1.000 (+/− 2.16E-6) before to 1.000 (+/− 2.34E-6) after the knockout.

Comparing the probabilities that the model assigned to the two groups (capsule knockout vs. control knockout) showed that the mean probability was significantly lower in the capsule knockout group than in the control group (p value 4.66E-10, Wilcoxon paired t-test).

### Removal of VFs resulted in a virulence prediction change in some strains

Similar to the capsule locus knockout, *in-silico* knockout of the entire set of 155 known VFs (mean removal of 152,203 bp +/− 9,981 bp) was performed on the 436 genomes of strains for which initial classification had predicted invasiveness with a high level of confidence (≥0.99). Following this knockout, ML classification using the SP 32k approach found that the majority (n = 230/436, 52.8%) of these strains were now predicted to have a carrier phenotype (with a classification threshold of 0.5). The mean probability of invasiveness assigned by the model to the 436 strains went from 0.999 ± 0.001 before to 0.468 ± 0.416 after the knockout.

As a control, the same number of bp was removed from each of the 436 genomes but from randomly selected contigs. Following this removal, ML classification found that a much smaller proportion (n = 13/436, 3.0%) of these strains were now predicted to have a carrier phenotype (Figure 2B). Note that because the control bases were removed randomly, some relevant genome portions may have been removed by chance; indeed, on average, 6% of the bases removed affected a known VF (data not shown). The mean probability of invasiveness assigned by the model to the 436 strains went from 0.999 ± 0.001 before to 0.969 ± 0.129 after the knockout, indicating a more limited removal effect (Figure 2B).

Comparing the probabilities between the knockout and control groups showed that the mean probability of invasiveness assigned by the model to the knockout group was significantly smaller than that of the control group (p value 7.12e-72, Wilcoxon paired t-test).

### Knockout of individual genes identified unstudied VFs

Next, to verify learning of the ML model, all 2,808 *N meningitidis* genes were individually knocked out in all 436 genomes for which invasiveness was predicted with high confidence (≥0.99). For 306 (the relevant genes) of these 2,808 genes, the prediction delta was above the 95^th^ percentile of the prediction deltas in the same genome, and this was true for at least 50 genomes. Of the 306 relevant genes identified, 44 were part of the 155 genes in the known VF list, a significant overlap (p value 4.15e-10, Fisher’s exact test). Some of the 306 relevant genes were not annotated in PubMLST;^2^ therefore, we restricted the number of relevant genes to 291 in the subsequent analysis (Table S2).

Database for Annotation, Visualization, and Integrated Discovery (DAVID)^38^ analysis for functional annotation and enrichment of these 291 relevant genes revealed that genes in four annotation clusters were present significantly more often than would be expected at random: cluster 1 contained genes involved in adenosine triphosphate (ATP) and nucleotide binding (enriched by a factor of 4.52), cluster 2 contained C-terminal helicases (enriched by a factor of 2.55), cluster 3 contained P loop containing genes involved in nucleoside triphosphate hydrolase (enriched by a factor of 2.24), and cluster 4 contained genes involved in the ligation of amino acids for protein biosynthesis (enriched by a factor of 1.99) (Table S3). The top-ten genes with the highest mean delta ranking from these in silico knockout experiments are presented in Table 2; four (*siaA*, *siaB*, *siaC*, and *lbpA*) were already known VF genes.^39^ The other six are potential VF genes.

**Table 2:** Annotation of the top-ten ranking potential virulence factor (VF) genes identified through the in silico knockout approach

Rank	Locus ID^a^ in *Neisseria meningitidis*	Gene symbol	Protein name	MC58 (serogroup B)^b^	FAM18 (serogroup C)^b^	Reference	Known VF gene^39^
1	NEIS0054	*cssA, siaA, synA, synX, neuC*	N-acetylglucosamine-6-P 2-epimerase	NMB0070	NMC0054		Yes
2	NEIS0053	*cssB, siaB, synB*	CMP-N-acetylneuraminic acid synthase	NMB0069	NMC0053		Yes
3	NEIS1464	*secA*	preprotein translocase subunit SecA	NMB1536	NMC1464	In *Escherichia coli, secA* is part of the secretory pathway to deliver virulence factors to the extracellular environment;^40^ it is also the first gene of an operon with many different promoter sequences.^41^	No
4	NEIS0044		oligopeptide transporter (OPT) family	NMB0060	NMC0044	Is part of the capsule locus	No
5	NEIS1191	*ygfZ*	tRNA-modifying protein	NMB1024	NMC1191	Plays a role in iron–sulfur metabolism^42^ and contributes to secretion of CNF1 in *E. coli* OMVs.^43^	No
6	NEIS2113	*tamB*	putative periplasmic protein	NMB2135	NMC2113	Role similar to BAM complex.^44^ Part of the TAM autotransporter assembly complex, which functions in translocation of autotransporters across the outer membrane.^45^	No
7	NEIS1468	*lbpA*	lactoferrin-binding protein A	NMB1540	NMC1468		Yes
8	NEIS0418		putative cytochrome c-type biogenesis protein	NMB1803	NMC0418	Cytochrome *c* biogenesis assembly and maturation is critical for virulence of *Bacillus anthracis*.^46^	No
9	NEIS0052	*cssC, siaC, synC*	N-acetylneuraminic acid synthase	NMB0068	NMC0052		Yes
10	NEIS1645	*fixN*	cbb3-type cytochrome *c* oxidase subunit I	NMB1725	NMC1645	Involved in aerobic energy generation and overexpressed in MC58 in invasive conditions.^47^	No

Ranking is based on effect size.

BAM, β-barrel assembly machinery; OMV, outer-membrane vesicle; OPT, oligopeptide transporter; TAM, translocation and assembly module; tRNA, transfer ribonucleic acid; VF, virulence factor.

a Based on data in the PubMLST Database.^2^

b Locus code in specific *N. meningitidis* strains.

The first is *secA*, which in *N. meningitidis* is the first gene of an operon with three promoters (type I, II, and III).^41^ The translocase that *secA* encodes is part of the general secretory pathway delivering VFs to the extracellular environment for interaction with the host,^40^ and is thereby already known to be indirectly involved in virulence. The second, with locus ID NEIS0044, encodes an oligopeptide transporter. Little is known about this gene except that it flanks the capsule locus,^48^ which encodes the major *N. meningitidis* VF. The third, *ygfZ*, encodes a transfer ribonucleic acid (tRNA)-modifying protein that plays a role in iron–sulfur metabolism^42^ and contributes to the secretion of cytotoxic necrotizing factor 1, a VF, in outer-membrane vesicles (OMVs) of *Escherichia coli*.^43^ Whether it plays the same role in *Neisseria* has not been investigated. The fourth, *tamB*, encodes the inner membrane-anchored, periplasmic protein TamB, which is part of the translocation and assembly module (TAM) complex. The precise role of the TAM complex remains unclear, but it has been suggested that it has a role similar to the β-barrel assembly machinery (BAM) complex,^44^ which plays a role in the translocation of autotransporters across membranes.^49^ The fifth, with locus ID NEIS0418, encodes a putative cytochrome c-type biogenesis protein. Cytochrome *c* assembly and maturation are critical for the virulence of *Bacillus anthracis*^46^ and were found in *Neisseria* to be involved in biofilm formation, a growth form that protects bacteria during infection.^50^ The sixth, *fixN*, which encodes the cbb3-type cytochrome *c* oxidase subunit I, is involved in aerobic energy generation and is overexpressed in the *N. meningitidis* strain MC58 under invasive conditions.^47^ Based on the known biological functions of the six potential VF genes highlighted here, their role as VF genes is highly plausible.

### The bag-of-words classification and knockout approach for knowledge extraction are more informative than the state-of-the-art methodology in our datasets

We constructed phylogenetic trees based on gene presence or absence for both problems to compare our approach with the current state-of-the-art methodology. The phylogenetic trees are presented in Figure S1. The distance on the trees represents the similarity of genomes in terms of variety of gene sets. We then colored the genomes for their classification label to check whether they were clustering on the tree.

For the capsule classification, we observed defined groups of B and non-B genomes next to each other on the tree. This is expected, as from the literature,^51^ we know that some genetic backgrounds (sequence types and clonal complexes) are associated with a capsule type, even if the capsule locus does not reflect the evolution of *N*. *meningitidis*, as it is subject to recombination events. We also observed a few mixed clusters, that may correspond to a genetic background shared between B meningococci and other capsule groups (e.g., cc-11 is shared between MenB, MenC and MenW).^52^ Rarely, we observed points of different color into homogeneous clusters. These may be cases of capsule switching where the capsule operon recombined.^53^

For the disease classification, a more heterogeneous situation was observed, as the two labels were present in the majority of clusters. Also in this case, the genetic background has been associated with carrier and invasive states in the literature,^54^ identifying clonal complexes more prone to carrier or invasiveness. However, usually, an invasive state was preceded by a carrier state in the nasopharynx, thus, the changes that allow the escape in the blood would not dramatically change the genome, but more likely change a few specific loci.

We then performed random forest classification^31^ on the table reporting the presence or absence of genes in each genome for the carriage/invasive problem, where the number of genomes was suitable for gene presence or absence retrieval from PubMLST.^2^ The accuracy obtained with the test set for the classification was 84.2%. Our model is, therefore, outperforming the classification based on genes’ presence or absence.

Word analysis with Shapley Additive exPlanations (SHAP) or feature importance can sometimes help to derive insights in classification. To compare with the knockout approach, we also performed SHAP analysis. We plotted the distribution of SHAP values for the most important words in the dictionary, for the capsule task (Figure S2A) and the disease task (Figure S2B). As can be seen from the plot, some words influence the classification process. An example is the word TCAACTA: a high value associated with this word pushes the model output to classify the genotype of the Capsule B group, while low values seem linked to other genotype groups. The most important words from the SHAP analysis did not overlap with any transcription factor binding sites reported in public repositories. The reason for this is probably that the words might be several bases longer or shorter than transcription factor binding sites and thus are not recognized in the search. In contrast, the knockout experiments were designed explicitly to remove multiple words simultaneously, related to regions of interest in the genome. Therefore, we conclude that for this particular task of biological knowledge extraction, the knockout analysis is more informative than the word importance analysis, due to the multivariate role of words in the classification models, that cannot be observed in univariate analysis, i.e., when inspecting single words.

To compare genome similarity within and between classes, principal component analysis (PCA) was performed, and the first two principal components were visualized in scatterplots. In the case of the capsule task (Figure S3A), the genomes belonging to different classes were slightly misaligned, which indicated that the genotypes were already divided into the two phenotype classes to a certain extent. In contrast, in the case of the disease task (Figure S3B), the two classes almost completely overlapped, indicating that the classification was more difficult. Nonetheless, the ML model was able to pick up relevant patterns in both cases as the knockout experiments showed that, in the capsule case, the patterns corresponded to words belonging to the capsule locus; in the disease case, the knockout experiments showed that the patterns picked correlated with the presence of the virulence factors.

## Discussion

We have shown for the first time how an ML model can be used to classify bacterial phenotypes based on their entire genomic sequences. The ML model we developed consists of tokenizing genomes with SP or k-mers, encoding them as bag-of-words histograms, and then analyzing the entire encoded genomes with a downstream ML model for classification. We tested the model by classifying *N. meningitidis* genomes for capsule group B assignment and disease phenotype and obtained very high classification accuracies. Subsequently, we verified whether the ML model was indeed learning by determining whether predicted potential VF genes had a known biological function that meant a role as a VF gene was plausible.

The mean length of the *N*. *meningitidis* genomic sequences was, in accordance with the literature,^18^ around 2.2 Mbp, i.e., too large to be used as input for an ML model. Inspired by the approach proposed for promoter region and chromatin-profile prediction in human genomic sequences,^6^ we employed SP’s Byte Pair Encoding algorithm to generate a vocabulary that segmented entire *N*. *meningitidis* genomes into words. Using this ML model, we were able to classify *N. meningitidis* strains for either capsule B or disease phenotype with very high accuracy (99.6% and 90.2%, respectively). Of the two tokenization approaches applied, the SP approach performed better than the k-mer approach. One possible explanation for this result is that SP vocabularies can encode longer words than k-mer. For example, the 32k SP vocabulary had a mean word length of 17 (mode 8), and about 66% of words (21,121) were longer than eight bases. The k-mer vocabulary, on the other hand, had limited word length as the vocabulary size grew exponentially with k-mer length, so that greater lengths were computationally unfeasible. In addition, the k-mer approach uses all possible words, so it will also include words that have no impact on the classification. While both SP and k-mer approaches resulted in a very high performance, SP obtained better results using only one-tenth (capsule task) and one-third (disease task) of the vocabulary size needed by the best k-mer approach, hence requiring less computational resources. Therefore, we used SP for subsequent analyses.

The knockout experiments showed that removing the capsule locus almost always resulted in a change in the capsule prediction from B to non-B; this was statistically significant compared with removing a random region of the same length. This aligns with biological intuition, because the non-B group comprises various capsule types, while the B signal is very specific. Similarly, the knockout experiments that removed 155 VFs resulted in most cases in a change in the disease prediction, although the effect was not as clear as for the capsule task. Moreover, the controls also sometimes showed a change in prediction. So, it appeared that the random knockout of such large numbers of bases did not always target only genetic regions that were not relevant to the invasive phenotype. Another explanation might be that some of the strains were misclassified or that their classification was based on genome regions not meaningful for the specific problem. Similarly, even though the change in prediction was significantly more frequent when the known VFs were removed, it was not always observed, suggesting that additional VFs might be active in those genomes, or that loci cooperate. These observations probably occurred because the list of VFs known today, although based on state-of-the-art biological knowledge,^39^ is not comprehensive, and factors other than VFs may also affect invasiveness.

The ML method resulted in very high performance for both a classification that was strictly genetic (capsule group B assignment)^39^ and for a classification that was partly genetic and partly influenced by a host’s immunological response (disease phenotype).^55^ This suggests that our approach enabled joint consideration of genetic information as well as the underlying regulation and interplay. Experimental evidence will need to confirm that the underlying regulation and interplay were indeed considered.

In addition to applying the ML model for the classification of genomes, we verified that the ML model was indeed learning by using the model to identify potential new VFs and determining whether their role as a VF was plausible. Upon knocking out 2,808 genes, of the 306 relevant genes ranking highest for disease phenotype prediction, 44 were known VF genes. For 291 of the relevant genes, PubMLST data were available; these data indicated enrichment in four clusters. The first cluster contained genes involved in ATP or nucleotide binding, essential for nutrient acquisition and for secretion of toxins and antimicrobial agents and hence essential for invasion of a host.^56^^,^^57^ The other clusters contained genes involved in C-terminal helicases, P-loop-containing nucleoside triphosphate hydrolases, and ligation of amino acids for protein biosynthesis. While protein biosynthesis is a general pathway that is not specific for virulence, activation of this pathway is needed for adaptation to a new environment, such as during invasion. Indeed, genes involved in the biosynthesis of amino acids and proteins were found to be upregulated during the growth of *N. meningitidis* in the blood.^58^ Experimental evidence is needed to verify that genes in these last clusters also affect virulence.

The ML method we have developed may be a useful tool for the identification of genes that potentially contribute to a specific phenotype, in this case virulence. Other methods have been used previously to systematically identify *N. meningitidis* genes. One is the combination of open reading frame prediction and whole genome homology searches that became feasible once whole genomes could be sequenced. That method has been successfully applied to identify genes from various *N. meningitidis* strains by comparing them with sequences from other species.^18^^,^^59^^,^^60^^,^^61^^,^^62^ Those methods did not, however, allow for the systematic identification of genes involved in a specific process or phenotype. A more recent method is the analysis of entire transcriptomes under various experimental conditions, which enables the identification of genes involved in specific processes.^63^ An older method that has been updated is signature-tagged transposon mutagenesis screening, which can identify genes involved in a specific phenotype.^64^ The advantage of our ML method is that it identifies genes that are potentially involved in a specific phenotype without requiring laboratory experiments, thus providing an efficient pre-screening tool.

The objective of this analysis of potential VF genes was to evaluate the method and verify the biological significance of genomic regions that have the greatest impact on classification. Therefore, the results obtained were not intended to precisely clarify the invasive phenotype. However, in future work our ML method could be modified to generate a complete description of the genes important for classification, assisting the next generation of GWAS.

To our knowledge, this is the first ML method for the classification of bacterial strains based on their genomes. This method may in future be useful for strain characterization, population genomics, monitoring antibiotic resistance, and outbreak investigation. We showed that in *N. meningitidis*, the ML method not only had a high level of accuracy in two different classification tasks but can also be used to identify potential new VF genes. The ML method could thus also be used for pre-screening genes that may be linked to a specific phenotype, to reduce the number of subsequent laboratory experiments required.

### Limitations of the study

Our study has some limitations. First, having very good classification performances does not necessarily mean that the ML model was able to identify biologically meaningful information, although our results indicate this to some extent. Laboratory knock-in and knockout experiments should be performed to verify the role of the potential VFs. Second, our work is based on the bag-of-words approach, which purposely discards positional information, meaning that some information about how the genome is structured is lost, such as the positional dependency of regulatory elements. Currently, incorporating positional information in the bag-of-words approach would defeat its strength, namely that it is computationally less costly than alignment-based methods. Until future increases in computational power allow for positional information to be included, the bag-of-words approach is a valuable tool.

## STAR★Methods

### Key resources table

**Table:** 

REAGENT or RESOURCE	SOURCE	IDENTIFIER
**Deposited data**		

25,959 *N. meningitidis* genomes	PubMLST^2^	https://pubmlst.org 25,959 non-complete *N*. *meningitidis* genomes with sequence length ≥1.5 Mbp

**Software and algorithms**		

All code for preprocessing, tokenization, vectorization, training, and knock-outs	This paper	https://github.com/mbodini/genome-predictor
BigBird	Zaheer et al.^6^	https://github.com/google-research/bigbird
SentencePiece	Kudo et al.^29^	https://github.com/google/sentencepiece
LightGBM	Ke et al.^65^	https://github.com/microsoft/LightGBM
Random Forest	Breiman^66^	https://cran.r-project.org/web/packages/randomForest/index.html
Term Frequency-Inverse Document Frequency (TF-IDF)	Leskovec et al.^67^	https://github.com/scikit-learn
Shapley Additive exPlanations (SHAP)	Lundberg et al.^68^	https://github.com/shap/shap
Principal component analysis (PCA)	Pearson^69^	https://github.com/scikit-learn

### Resource availability

#### Lead contact

Further information and requests for resources should be directed to and will be fulfilled by the lead contact, Margherita Bodini (margherita.x.bodini@gsk.com).

#### Materials availability

This study did not generate new unique reagents.

#### Data and code availability

• All data reported in this paper will be shared by the lead contact upon request.
• All original code has been deposited at: https://github.com/mbodini/genome-predictor and is publicly available as of the date of publication. DOIs are listed in the key resources table.
• Any additional information required to reanalyze the data reported in this paper is available from the lead contact upon request.

### Method details

#### Genome datasets

A set of 25,959 non-complete *N*. *meningitidis* genomes with sequence length ≥1.5 Mbp were downloaded in FASTA format from the PubMLST website^2^ on June 16, 2021. The genomes analyzed are available in Table S4A (capsule task) and Table S4B (disease task). The sequences were represented using the four bases (A, C, T, and G) and the International Union for Pure and Applied Chemistry (IUPAC) codes (such as N, K, W, Y, R, and S) for bases not unequivocally identified. From these genomes, we extracted two datasets: the first dataset (the capsule dataset) contained 25,290 genomes for which the “capsule_group” field (a genotype based on both serogroup and genogroup data) was not null and not “discrepancy.” In this dataset, the 10,166 genomes with capsule type B were labeled 1, and the other 15,124 genomes were labeled 0. The second dataset (the disease dataset) contained 20,442 genomes whose “disease” field was not null. In this dataset, the genomes annotated as “carrier” were labeled 0, and those annotated “invasive (unspecified/other),” “septicaemia,” “meningitis and septicaemia,” and “meningitis” were labeled 1.

We noticed that the resulting datasets had a bias in the quality of the assemblies, most likely due to the sequencing method used to obtain them. Specifically, the distribution of the genome proportion covered by the ten longest contigs (hereafter referred to as the “contig length distribution”) was biased toward strains with capsule type B (see below figure) and carrier strains (see below figure). This bias could mislead the model to predict capsule type (respectively invasiveness) by detecting this pattern while neglecting biological (and thus more relevant) patterns. To eliminate this bias, we down-sampled genomes from the predominant class such that their number equaled that of the minority class while at the same time ensuring that the samples were drawn from a close approximation of the contig length distribution of the minority class. The approximate distribution was obtained by first calculating the genome proportion covered by the ten longest contigs for all genomes in the dataset, then by binning resulting values with k-means clustering, and finally by sampling an equal number of genomes for the two classes for each cluster. The final datasets, comprising 19,498 and 9,402 genomes for the capsule and disease datasets, respectively, were thus balanced for the two classes and had similar quality distributions, as can be seen by the overlap of the curves in below figure.

**Figure:**
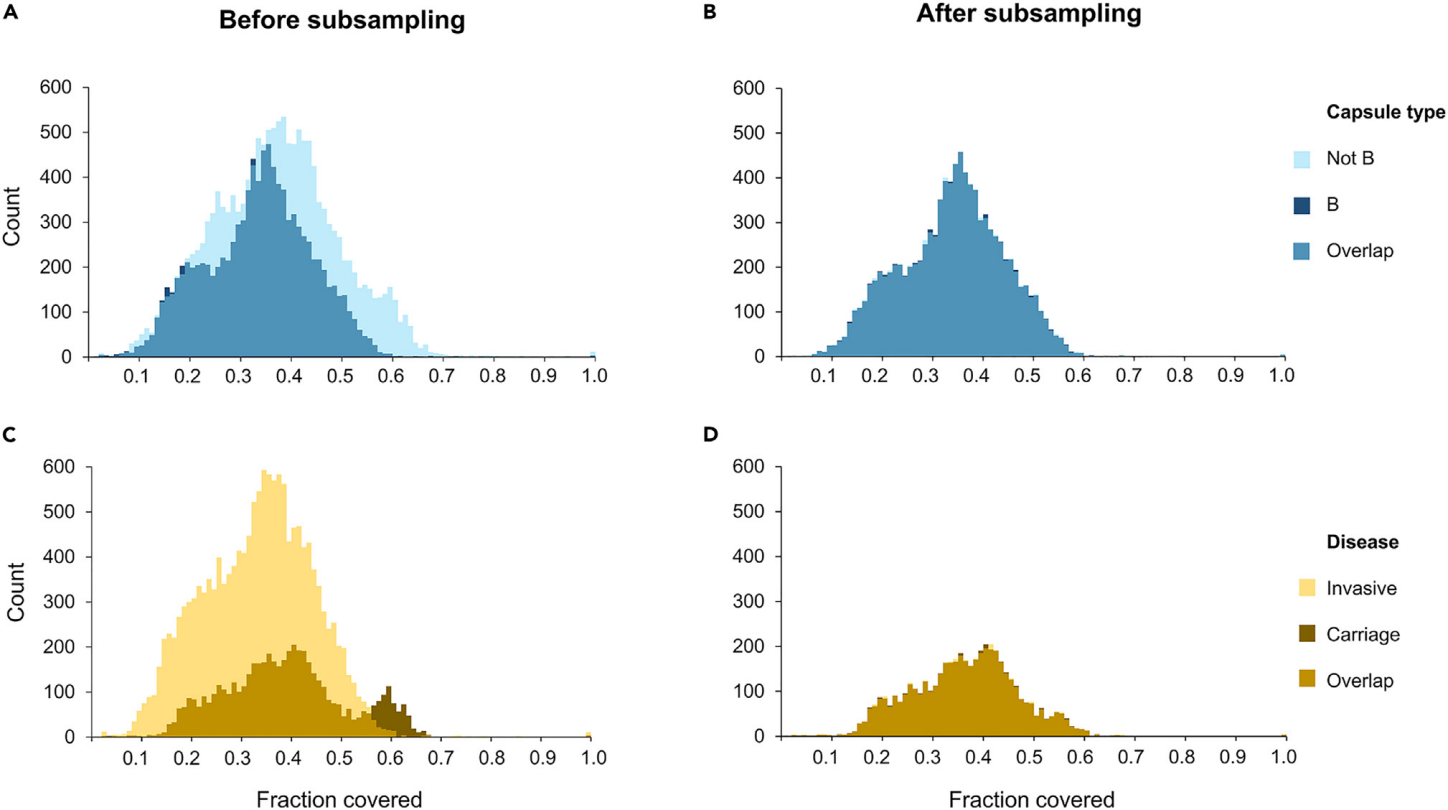
Genome sets after resampling to reduce bias in datasets Histograms of the fractions of each genome in the dataset covered by the 10 longest contigs. (A) The 25,290 genomes of the capsule dataset, light blue bars indicate capsule group non-B strains, dark blue bars indicate B strains, medium blue bars indicate where light and dark bars overlap. (B) The 19,498 genomes of the reduced dataset after resampling capsule group B samples, bars are color-coded in the same way as for (A). (C) The 20,442 genomes of the disease dataset, light yellow bars indicate invasive strains, dark brown bars indicate carrier strains, medium brown bars indicate where light and dark bars overlap. (D) The 9,402 genomes of the reduced dataset after resampling invasive samples, bars are color-coded in the same way as for (C). The y axis indicates the number of genomes; the x axis indicates the fraction of the genomes covered.

The two datasets were each split in three by randomly extracting 500 and 1,000 samples for the validation and the test sets, respectively. The remaining genomes were used as training sets (see below table). The validation sets were used to select the model’s hyperparameters; the test sets were preserved for independent performance evaluation.

**Table:** Genome datasets divided into training, validation, and test sets

Task	Set	Genomes from carrier strains	Genomes from invasive strains	Total
Capsule classification	Training	8,999	8,999	17,998
	Validation	250	250	500
	Test	500	500	1,000
				19,498

Task	Set	Genomes from capsule group B strains	Genomes from non-B capsule group strains	Total
Disease classification	Training	3,951	3,951	7,902
	Validation	250	250	500
	Test	500	500	1,000
				9,402

Before the vocabulary creation using either tokenization method, all characters not unequivocally identified were masked with “X”.

#### Byte pair encoding

Byte pair encoding is an algorithm to create token vocabularies from a corpus of sequences. It starts by creating an initial vocabulary where tokens are single characters in a given alphabet. Then, the most frequent pair of adjacent tokens is merged into a new token, which is added to the vocabulary. Finally, all the occurrences of the two merged tokens are replaced by the new token in the corpus. This procedure is iterated to expand the vocabulary until a desired size is reached.

#### Vocabulary creation with SP

Tailoring the BigBird procedure,^6^ we created a training corpus for SP containing 1,000,000 subsequences, with a length between 500 and 1,000 consecutive bp, randomly extracted from all genomes in the training set. SP generated the vocabulary using Byte Pair Encoding,^70^ by repeatedly adding the most frequent word pairs present in the corpora as a new vocabulary term. Starting from a vocabulary containing the four bases A, C, T, and G, along with X to represent unknown characters, SP iteratively added new words to the vocabulary by merging the two most frequent words until the desired vocabulary size was reached. Any token with an “X” was subsequently removed from the vocabularies. In this study, we experimented with six vocabulary sizes (2k, 4k, 8k, 16k, 32k, and 64k).

#### Vocabulary creation with k-mers

For a given k and an alphabet of four characters (A, T, C, and G), the number of possible k-mers is 4^k^. We constructed six different k-mer vocabularies by varying k from 3 to 8.

The overall dimension of the vocabulary was obtained with the formula:

$$V\left(k\right)=\sum_{i=3}^{k}4^{i}$$

Note that for k = 8, we obtain a vocabulary with 87,360 words: $V\left(k\right)=\sum_{k=3}^{8}4^{k}$.

#### Bag-of-words approach

A bag-of-words approach transforms any linearly ordered set of words, e.g., a sentence, a document, or, as here, a sequence, into an unordered set, hence the name bag-of-words.^71^ Each sequence can be seen as a sentence, represented in a simplified way as the bag (multiset) of its words, regardless of relative position, direction, and order, but keeping multiplicity. We developed a bag-of-words approach to encode, after SP or k-mer tokenization, entire bacterial genomes.

#### TF-IDF vectorization

After creating the vocabularies, we transformed the N genomes into a $D\in R^{N\times V}$ matrix, where V is the vocabulary size. The (i, j)-th matrix entry, indicated as $d_{ij}$, was obtained as follows:

$$d_{ij}=tf_{ij\;}\times \log\left(\frac{N}{df_{i}}\right)$$

Where tf_ij_ is the term frequency (how many times word i appears in genome j), and df_i_ is the document frequency (how many times word i appears across the entire corpus). This representation is called the Term Frequency-Inverse Document Frequency (TF-IDF)^67^ and is used here to represent a genome in numerical form throughout our experiments. In short, genomes are represented as histograms of size V, where each bin corresponds to a word, with each word’s numerical value being the number of occurrences in the genome, normalized by its relative frequency across the training set. As the sequencing direction of contigs cannot be known without alignment, we achieved directionality invariance by tokenizing each contig in both directions and summing up the two histograms. We further summed all of the contig histograms to obtain a fixed-length representation of each genome, which was used as the input for the downstream classifier.

#### Gradient Boosting Machines and lightGBM

Gradient Boosting Machines (GBMs) belong to the family of ensemble methods, i.e., methods that aggregate a pool of “weak” (in terms of performance) models such that the combined model performs better than the single models in isolation. In particular, each component of a GBM is trained sequentially to minimize the error residual of the previous. Predictions can be obtained by a weighted average of each component’s prediction. lightGBM is a library for Gradient Boosting Trees,^65^ that is, GBMs that ensemble multiple Decision Trees. It offers a broad series of optimizations and facilities to train Gradient Boosting Trees on large datasets. Currently, it is one of the best-performing GBM libraries available and is widely adopted by the ML community.

#### Model evaluation

The GBM was chosen as the classifier, specifically the lightGBM implementation,^65^^,^^72^ as it is a general-purpose classifier that has repeatedly achieved strong performances in a wide range of tasks.^16^ The classifier takes as the input an entire *N. meningitidis* genome encoded as a bag-of-words and provides as the output a real value in the range 0–1, representing the probability that the genome is of capsule B (for the capsule task) or invasive (for the disease task). To identify the best model, we randomly sampled 50 unique combinations of hyperparameters from prespecified distributions with a randomized hyperparameter search^73^ (Table S5). For the SP approach, six vocabulary sizes were evaluated: 2k, 4k, 8k, 16k, 32k, and 64k. For the k-mer approach, the genome was segmented in k-mers: only 3-mers (vocabulary size 64), 3- and 4-mers (vocabulary size 320), 3- to 5-mers (vocabulary size 1,344), 3- to 6-mers (vocabulary size 5,400), 3- to 7-mers (vocabulary size 21,824), and 3- to 8-mers (vocabulary size: 87,360).

For each hyperparameter combination, we instantiated the corresponding classifier, trained it on the training set, and evaluated its performance on the validation set with the metrics accuracy, area under the curve of the receiver operating characteristic (AUROC), and Matthew’s correlation coefficient. The model with the highest validation accuracy was selected, trained again using the training and validation partitions as training sets, and evaluated on the test set. All experiments were carried out with a fixed and known random seed to ensure reproducibility. The pipeline is illustrated in below figure.

**Figure:**
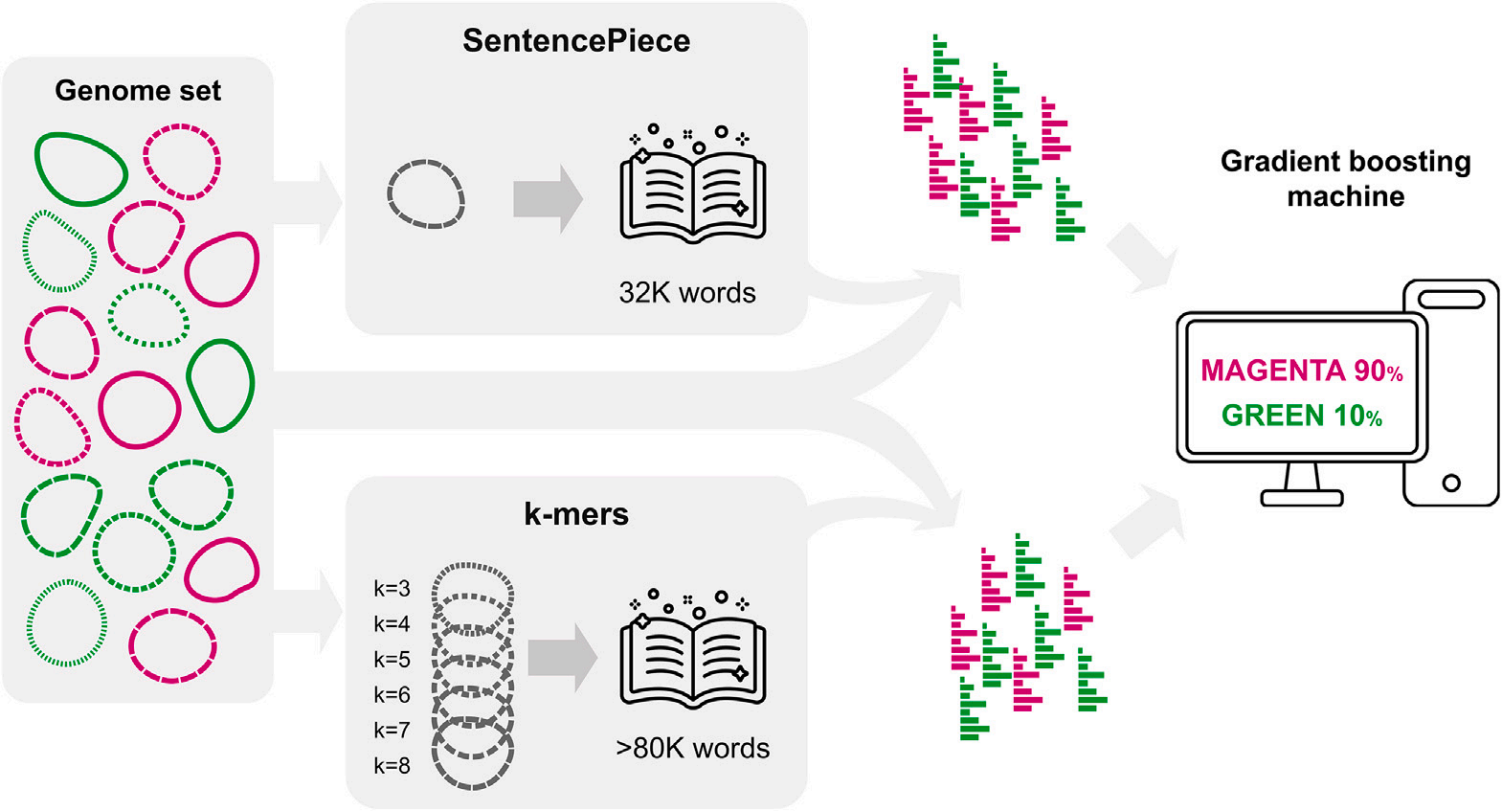
Pipeline from genomes to predicted classification Shown are the tokenization by the SentencePiece and k-mer approaches, encoding the tokens as bag-of-words histograms, and the machine learning method. Magenta and green indicate the genomes of the two different classes (capsule B vs. non-B capsule, or invasive vs. non-invasive) per task. The genomes were fragmented into words for both approaches, and vocabularies were created accordingly. Then, each genome was presented as a histogram, based on the occurrence of words in the dictionary. As an example, the classification output for two complementary probabilities for the imaginary ‘magenta’ and ‘green’ classes is depicted on the screen on the right side of the figure.

#### Phylogenetic trees

The information on the presence or absence of genes was downloaded from the PubMLST website^2^ for all the genomes used in the two classification tasks, together with the Newick tree files for each genome set. Phylogenetic trees were constructed using ape package in R suite.

Classification was performed in R suite with the randomForest package.^74^ After the removal of not-informative columns, we used the same training set used for our bag-of-words approach for learning, then the validation set for parameter (mtry) optimization, and finally, the test set for performance evaluation.

#### SHAP analysis

To study the feature contributions to the model predictions, we used SHAP, which measures the importance of the features used by an ML model.^68^ SHAP is based on Shapley values, which have been developed in game theory to assign individual credit from an aggregated outcome. Specifically, SHAP works by decomposing an output prediction into a sum of individual contributions given by the single features. Each feature is then assigned a value representing its relationship with the output: positive (resp. negative) values indicate that the output prediction is positively (resp. negatively) correlated with the prediction. We ran SHAP on the single test predictions and then aggregated the output into a single plot showing, for each feature, one circle for each genome indicating the individual contribution made to the prediction of that genome.

#### PCA analysis

To compare genome similarity within and between classes and find possible biases, we used PCA. PCA is based on applying an orthogonal transformation to the data to derive a set of linearly independent variables called principal components.^69^ The most important principal components explain most of the variation in the original data and as such can be used to find explanatory patterns. Specifically, we applied PCA to the genome histograms and kept only the first two principal components, meaning that we transformed each genome into two coordinates. These coordinates were then plotted on a 2D plane and colored according to the genome class, to determine whether the data contained patterns that would bias the classification toward one class or the other.

#### In silico gene knockout

Knockouts of genes (or genomic regions) were used to determine whether the classifier predictions could be linked to the biological processes underlying each classification task and, if so, to what extent. Knockouts were performed on genomes where the classifier was highly confident (prediction ≥0.99) that they were capsule B (for the capsule task) or invasive (for the disease task). A knockout entailed masking a sequence of consecutive bases (a gene or genomic region of interest) with an equal number of “X” characters, which were then discarded before tokenization. This effectively altered the TF-IDF of the masked words and produced a different histogram for the genome. To rule out confounding effects, we created a control for each knockout by removing a different sequence (with the same number of bases) from the same genome.

For the capsule classification task, we knocked out the intergenic region between NEIS0044 and NEIS0068 (on average 25k bases) from all genomes where it was contained in a single contig (n = 32). As control, we removed from the same genomes an equal portion of contiguous bases taken from a randomly chosen contig.

For the disease classification task, as VFs play a role in invasiveness, we removed from a set of 436 genomes a panel of 155 non-consecutive loci (Table S6) for which a PubMLST annotation was available from a panel of 172 known VFs.^39^ As controls, we removed 155 non-consecutive loci (with the same number of bases) from random regions of the same 436 genomes.

For both the capsule and disease tasks, we recorded the prediction before and after removing the knockout and control loci. Finally, we used a Wilcoxon signed rank test (significance level alpha = 0.05) to establish whether the knockout predictions were significantly different from the control predictions.

#### Identification of unstudied VFs plus annotation

To identify relevant genes that influence the prediction of invasiveness, we further performed single-locus knockouts for 2,808 *N meningitidis* loci annotated on PubMLST.^2^ A locus was considered relevant if its prediction delta (the difference in prediction before and after knockout) on a single genome was above the (arbitrarily chosen) 95^th^ percentile and this was observed in ≥50 genomes. These criteria ensured that we considered all relevant genes that had a strong effect both on individual genomes and across genomes.

Each gene was then ranked by the number of times it satisfied both conditions across the 436 genomes. The set of relevant genes obtained was subsequently analyzed for enrichment in the list of 155 VFs (Table S6) (Fisher’s exact test, significance level 0.05) and DAVID.^38^ Functional annotations were based on the literature and pathway enrichment with DAVID’s Functional Annotation Chart and Functional Annotation Clustering with the lowest classification stringency for gene ontology (GO) enrichment. The significance threshold for the Benjamini–Hochberg adjusted p value on enrichment was 0.05.

### Quantification and statistical analysis

Statistical analyses were performed using custom python scripts. Details of all statistical analyses can be found above in the relevant subsections of the method details section. Sample number (n) and statistical methods used to assess differences between groups are indicated in the relevant subsections of the Results section.

## Supplemental information

Document S1. Figures S1–S3 and Tables S1, S3, and S5

Table S2. Annotation of the 291 relevant genes from the in silico knockout approach, related to Table 2

Table S4. List of the genomes analyzed in this study, related to STAR Methods A, the 19,498 genomes analyzed for the capsule task. B, the 9,402 genomes analyzed for the disease task.

Table S6. List of the 155 VF genes that were knocked out to test disease prediction, related to STAR Methods
