# Supplementary material for: Classification of Neisseria meningitidis genomes with a bag-of-words approach and machine learning
Source: iScience. 2024 Feb 16;27(3):109257. doi: 10.1016/j.isci.2024.109257 (PMC10910294; doi:10.1016/j.isci.2024.109257)
Supplement: Document S1. Figures S1–S3 and Tables S1, S3, and S5 [file mmc1.pdf]

iScience, Volume *27*

Supplemental information

Classification of *Neisseria meningitidis* genomes with a bag-of-words approach and machine learning

Marco Podda, Simone Bonechi, Andrea Palladino, Mattia Scaramuzzino, Alessandro Brozzi, Guglielmo Roma, Alessandro Muzzi, Corrado Priami, Alina Sîrbu, and Margherita Bodini

Supplemental information

Figure S1: Phylogenetic trees based on gene presence or absence, related to STAR Methods.

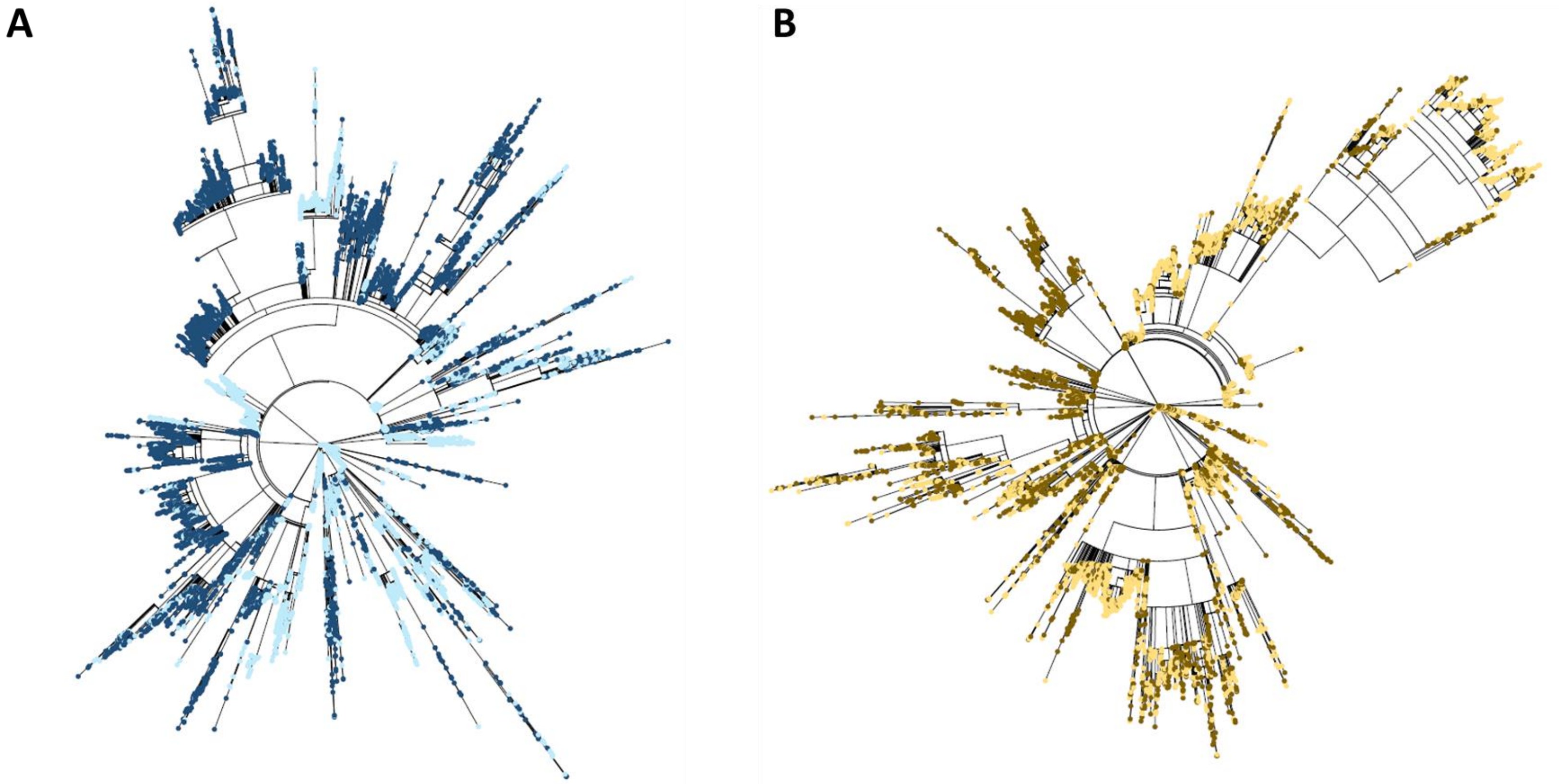

Phylogenetic trees based on gene absence or presence. **A**, Capsule classification **B**, Carriage/invasive classification.
Each circle represents one genome. Dark blue is capsule B, light blue is capsule non-B, yellow is invasive, brown is carriage.

Figure S2: SHAP analysis for classification, related to STAR Methods.

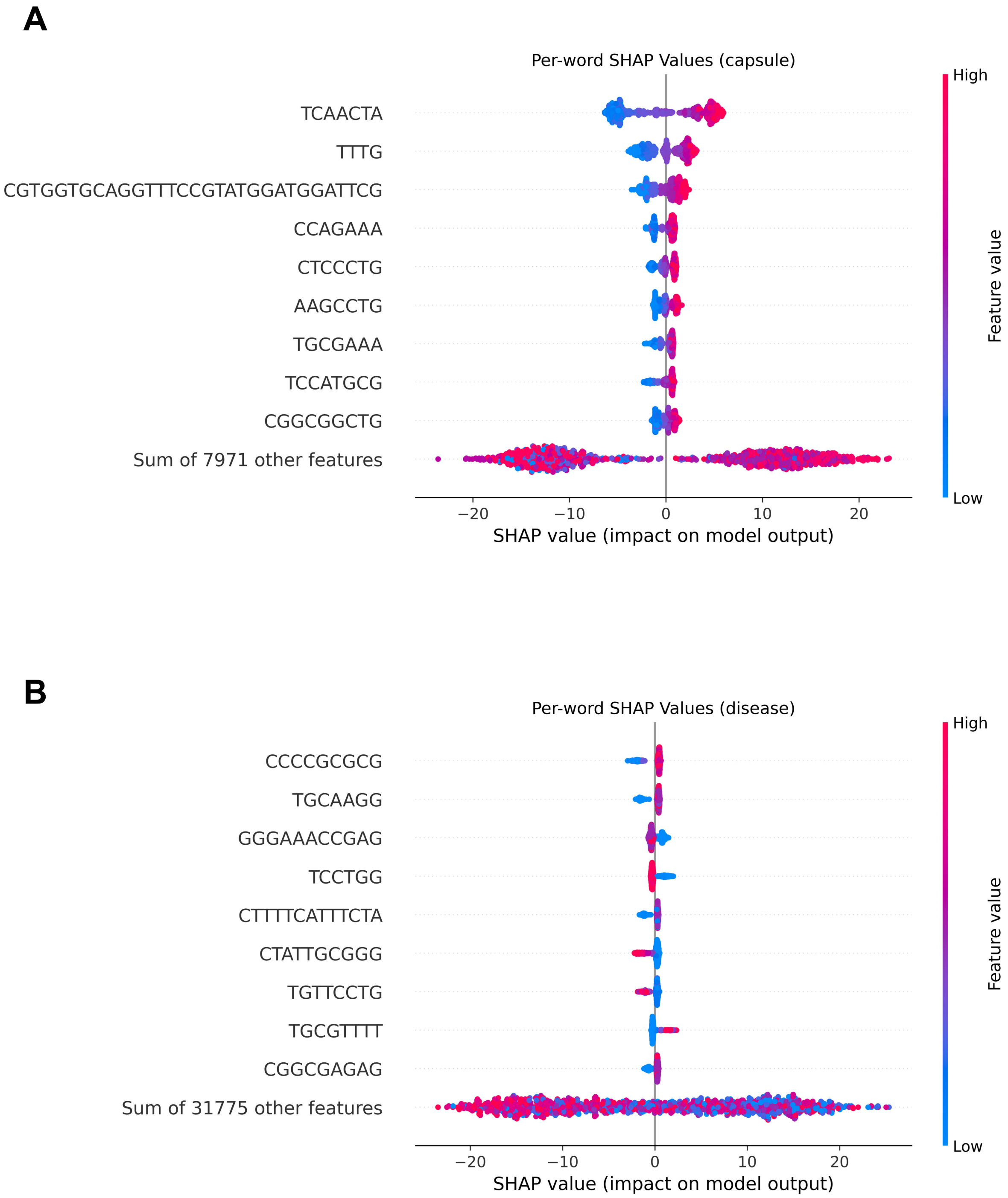

SHAP analysis of the classifiers for **A**, capsule task, **B** disease task. Each row is a word in the vocabulary, each circle represents a genome. For each word, genomes are colored based on whether the corresponding TF-IDF term in the data matrix has high (red) or low (blue) feature value.

Figure S3: PCA scatter plots to compare genome similarity within and between classes, related to STAR Methods.

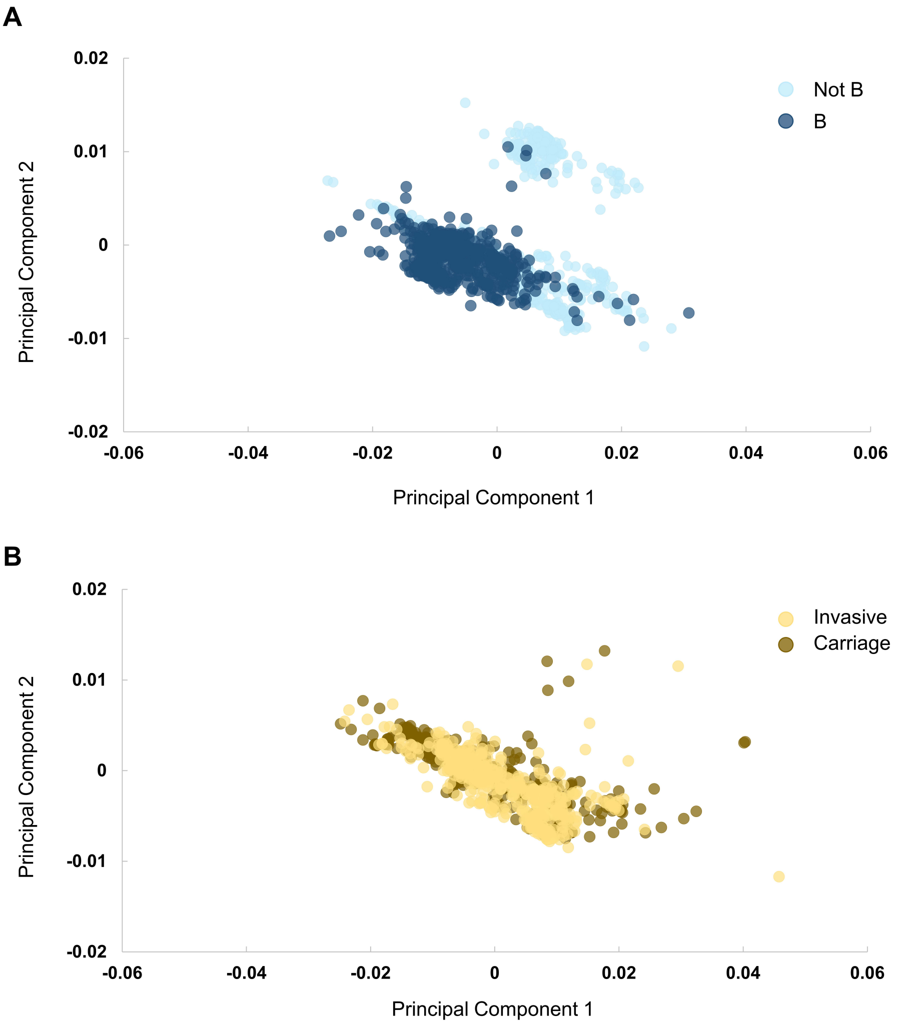

PCA scatter plots of **A**, the genomes used for the capsule task and **B**, the disease task. Each circle represents one genome. PCA, principal component analysis.

Table S1: Results of model evaluations, related to Figure 1.

Method	Vocabulary size	Capsule task			Disease task		
		Accuracy	AUROC	Matthew's correlation coefficient	Accuracy	AUROC	Matthew's correlation coefficient
k-mer	64	0.908	0.953	0.816	0.726	0.787	0.454
k-mer	320	0.946	0.979	0.892	0.784	0.847	0.569
k-mer	1,344	0.960	0.991	0.920	0.802	0.867	0.604
k-mer	5,440	0.976	0.996	0.952	0.812	0.872	0.624
k-mer	21,824	0.992	0.999	0.984	0.820	0.892	0.640
k-mer	87,360	0.998	1.000	0.996	0.830	0.890	0.661
SP	2,000	0.992	0.999	0.984	0.882	0.937	0.764
SP	4,000	0.998	1.000	0.996	0.884	0.944	0.768
SP	8,000	1.000	1.000	1.000	0.894	0.943	0.788
SP	16,000	1.000	1.000	1.000	0.896	0.958	0.792
SP	32,000	1.000	1.000	1.000	0.902	0.956	0.804
SP	64,000	1.000	1.000	1.000	0.894	0.950	0.788

Threshold used: 0.5. AUROC, area under the curve of the receiver operating characteristic; SP, SentencePiece

Table S3: Enrichment of the relevant genes in four annotation clusters, related to STAR Methods.

Annotation cluster	Enrichment score	Term	Number of genes	Percentage of the 291 relevant genes	p-value	Genes
1	4.52	GO:0005524 ATP-binding	72	24.8 %	3.74×10^{-8}	NMB1443, NMB0274, NMB1400, NMB0030, NMB1720, NMB1368, NMB0554, NMB0356, NMB1682, NMB0551, NMB0232, NMB1605, NMB0798, NMB1325, NMB0836, NMB1920, NMB1291, NMB1051, NMB1972, NMB1258, NMB1131, NMB1331, NMB1934, NMB1936, NMB0962, NMB0720, NMB1930, NMB1897, NMB1139, NMB1536, NMB1855, NMB0724, NMB0728, NMB2151, NMB2075, NMB1861, NMB0174, NMB0212, NMB1067, NMB0854, NMB1506, NMB1948, NMB0935, NMB1425, NMB1788, NMB1900, NMB0618, NMB2083, NMB2160, NMB1233, NMB1554, NMB0466, NMB0224, NMB0785, NMB1153, NMB1472, NMB0740, NMB1231, NMB0420, NMB1595, NMB0782, NMB1835, NMB0545, NMB0423, NMB2007, NMB1314, NMB1556, NMB1996, NMB0549, NMB1833, NMB1919, NMB1281
		KW-0067 ATP-binding	55	19.0 %	5.55×10^{-4}	NMB1443, NMB0274, NMB0030, NMB1368, NMB0554, NMB2011, NMB1682, NMB1325, NMB0836, NMB1920, NMB1972, NMB1131, NMB1331, NMB1934, NMB1936, NMB0962, NMB0720, NMB1930, NMB1897, NMB1139, NMB1536, NMB1855, NMB0724, NMB0728, NMB2151, NMB2075, NMB0174, NMB1067, NMB0854, NMB1506, NMB0935, NMB1425, NMB1788, NMB1900, NMB0618, NMB2083, NMB2160, NMB1233, NMB1554, NMB0466, NMB0224, NMB1153, NMB1472, NMB0420, NMB1595, NMB1835, NMB0545, NMB0423, NMB2007, NMB1314, NMB1556, NMB1996, NMB0549, NMB1833, NMB1919
		KW-0547 Nucleotide-binding	60	20.7 %	1.33×10^{-3}	NMB0274, NMB0030, NMB1643, NMB0554, NMB2011, NMB1682, NMB1325, NMB1920, NMB1972, NMB1450, NMB1131, NMB1331, NMB1934, NMB0766, NMB1936, NMB0962, NMB0720, NMB1930, NMB1897, NMB1139, NMB1536, NMB1855, NMB0724, NMB0728, NMB2151, NMB2075, NMB0174, NMB1067, NMB0854, NMB1506, NMB0138, NMB0815, NMB0935, NMB1425, NMB1788, NMB1900,

						NMB0618, NMB2083, NMB2160, NMB1233, NMB1476, NMB1554, NMB0466, NMB0224, NMB1153, NMB1472, NMB0420, NMB1199, NMB1595, NMB1835, NMB0545, NMB0423, NMB0947, NMB2007, NMB1314, NMB1556, NMB1996, NMB0549, NMB1833, NMB1919
2	2.55	IPR001650 Helicase, C-terminal	8	2.8%	7.30×10^{-5}	NMB0274, NMB1368, NMB1331, NMB0551, NMB1281, NMB2007, NMB1788, NMB1536
3	2.24	IPR027417 P-loop containing nucleoside triphosphate hydrolase	43	14.8 %	1.51×10^{-6}	NMB1443, NMB0274, NMB1400, NMB1643, NMB1720, NMB1368, NMB0356, NMB2011, NMB1067, NMB0232, NMB0551, NMB0798, NMB0138, NMB1948, NMB0815, NMB0935, NMB0836, NMB1788, NMB2160, NMB1051, NMB1233, NMB1554, NMB1258, NMB0785, NMB1153, NMB1472, NMB0740, NMB1231, NMB1199, NMB1331, NMB0782, NMB1934, NMB0766, NMB0545, NMB1936, NMB0962, NMB1314, NMB2007, NMB0549, NMB1536, NMB0329, NMB1919, NMB1281
4	1.99	KW-0030 Aminoacyl-tRNA synthetase	15	5.2 %	1.55×10^{-4}	NMB0174, NMB0030, NMB0466, NMB1595, NMB1835, NMB0854, NMB1506, NMB0720, NMB1930, NMB1897, NMB1425, NMB1833, NMB0724, NMB0728, NMB2083
		KW-0648 Protein biosynthesis	19	6.6 %	2.13×10^{-4}	NMB0174, NMB0030, NMB1643, NMB2138, NMB0466, NMB1595, NMB1835, NMB0854, NMB0766, NMB1506, NMB0138, NMB0720, NMB1930, NMB1897, NMB1425, NMB1833, NMB0724, NMB0728, NMB2083
		KW-0436 Ligase	26	9.0 %	2.46×10^{-3}	NMB1861, NMB0174, NMB0030, NMB0854, NMB1506, NMB0815, NMB1425, NMB1920, NMB2083, NMB1554, NMB0466, NMB0420, NMB1595, NMB1835, NMB0666, NMB0423, NMB0720, NMB1897, NMB1930, NMB1996, NMB1833, NMB1855, NMB0724, NMB0728, NMB2151, NMB2075

ATP, adenosine triphosphate; GO, Gene Ontology entry; IPR, InterPro entry; KW, UniProt entry; NMB, *Neisseria meningitidis* serogroup B gene; tRNA, transfer ribonucleic acid

Table S5: Random hyperparameter combinations used in the model selection, related to STAR Methods.

Combination	Maximum bin	Maximum depth	Number of estimators	Number of leaves
1	16	9	1,716	83
2	31	7	1,883	25
3	23	9	1,099	86
4	28	4	1,920	74
5	5	4	1,349	181
6	29	9	572	25
7	24	7	1,476	213
8	19	4	1,350	115
9	28	5	1,255	45
10	23	7	1,410	183
11	4	5	1,789	201
12	4	6	744	167
13	7	7	1,382	199
14	6	4	628	180
15	25	9	1,256	33
16	19	8	1,566	202
17	28	5	1,845	247
18	13	5	791	118
19	27	7	930	98
20	31	4	898	115
21	25	8	1,133	186
22	24	7	824	22
23	8	8	1,655	220
24	8	8	1,667	164
25	7	6	1,291	223
26	17	9	1,060	65
27	9	5	1,972	111
28	9	4	1,816	178
29	20	9	887	114
30	14	9	868	247
31	25	5	883	16
32	14	6	1,055	202
33	27	7	630	137
34	6	7	1,234	139
35	7	6	1,239	164
36	21	6	1,935	30
37	13	6	1,205	52
38	14	7	1,372	27
39	6	7	1,604	48
40	26	4	1,306	35
41	18	6	1,895	200
42	12	9	786	40
43	6	7	594	242
44	15	9	796	88
45	23	4	1,934	210
46	28	8	823	252
47	18	4	1,784	211
48	17	7	1,520	102
49	17	5	1,343	200

Combination	Maximum bin	Maximum depth	Number of estimators	Number of leaves
50	20	4	1,425	165
